# Sodium hydrogen sulfide (NaHS) ameliorates alterations caused by cisplatin in filtration slit diaphragm and podocyte cytoskeletal in rat kidney

**DOI:** 10.15171/jnp.2017.26

**Published:** 2017-02-06

**Authors:** Asiyeh Karimi, Forouzan Absalan, layasadat Khorsandi, Armita Valizadeh, Esrafil Mansouri

**Affiliations:** Cellular and Molecular Research Center, Department of Anatomical Sciences, Faculty of Medicine, Jundishapur University of Medical Sciences, Ahvaz, Iran

**Keywords:** NaHS, Cisplatin, Podocyte, Nephrin, Desmin

## Abstract

**Background:**

Hydrogen sulfide (H2S) has been shown to have a protective role in various kidney disorders.

**Objectives:**

This study investigated the molecular mechanism of NaHS (a H2S donor) in treating on the renal damage induced by cisplatin (CP).

**Materials and Methods:**

Thirty-two male rats were randomly divided into 4 groups: Normal control group (group A)‚ NaHS group (group B) which received 200 µg/kg/d (intraperitoneal injection; i.p.) for 15 days‚ CP group (group C) which rats were injected with CP (5 mg/kg, single dose, i.p.), and CP + NaHS group (group D) (5 mg/kg and 200 µg/kg, respectively, i.p.). Samples of urine and serum, tissue of kidney were collected for analysis after treatments for 15 days. Morphological changes were elevated under light microscope‚ protein expression of desmin and nephrin were determined by immunohistochemistry and western blotting and also malondialdehyde (MDA) level was determined by spectrophotometer.

**Results:**

Compared to the CP group, NaHS treatment mitigated histological damages, decreased 24-hour urine protein excretion, serum urea and creatinine as well as MDA level. NaHS treatment increased protein levels of nephrin. Moreover, NaHS treatment decreased protein levels of desmin.

**Conclusions:**

NaHS can ameliorate CP -induced renal damage in rats which is associated with the increase in nephrin protein expression, and the decrease in MDA level and desmin protein expression.

**Implication for health policy/practice/research/medical education:**

The anticancer drug cisplatin is known to have toxic side-effects on kidney. From the findings of the present study, we can conclude that, NaHS has an ameliorative effect against CP-inducedoxidative stress and renal damage through reduction of oxidative stress and alteration in expression of desmin and nephrin proteins.

## 1. Background

 Cisplatin (CP) is a well-known antineoplastic drug extensively used for treatment of different solid tumors, such as cervical, ovarian cancer and testicular cancer (1,2). However, it has been observed that CP accumulates in the kidney with a greater degree than other organs and so renal damage is the major dose- limiting side effect of this drug (2,3). The mechanism for this CP-induced renal injury has been intensively studied for years, and the results of the latest researches suggest that part of this renal cell injury is likely explained by inflammation, oxidative stress injury, and apoptosis (2). Oxidative stress will be induced through the imbalance between production of reactive oxygen species (ROS) and antioxidant systems for scavenging the reactive intermediates (4,5). As a result, podocyte injury such as DNA damage and apoptosis occurs and, proteinuria is developed after injuring podocytes. The specific structure of podocytes mainly depends on the expression of cytoskeletal system components and a number of specific proteins. These special protein molecules and the slit diaphragm (SD) proteins that regulate normal renal function are main portions of the podocyte barrier (4,6). One of the main filtration SD components produced by podocytes is nephrin protein. A mutation in gene this protein can lead to the loss of its expression, causing destruction of SD and significant proteinuria. Nephrin can be utilized as a key biomarker for early detection of acute glomerular podocyte injury as the decrease in its protein expression occurs earlier than ultrastructural changes in podocytes (6,7). Desmin protein is an intermediate filament of the cytoskeleton, which is secreted by podocytes during injury, whose its expression is remarkably correlated with podocyte injury (8,9). Two main interventional strategies for renal injury are prevention and treatment of podocyte injury and amelioration of proteinuria (9). Hydrogen sulfide (H_2_S) known as;the third gas transmitter after nitric oxide (NO) and carbon monoxide (CO), is generated at very levels in nearly all tissues or organs, such as brain, pancreas, liver and kidney (10). In mammalian systems, the production of this gas has been attributed to cystathionine β-synthase (CBS) and cystathionine γ-lyase, two main enzymes in cysteine biosynthesis pathway (11). In different cellular injury models, cytoprotective effects are observed for H_2_S at physiological concentrations (12). These effects are in part associated with the ability of H_2_S in neutralizing ROS, promoting relaxation of vascular smooth muscle, decreasing of apoptotic signaling, and reversibly modulating mitochondrial respiration (11,13). Topical administration of NaHS on kidneys can improve renal function and plays an important role in protection against renal ischemia (12,14). H_2_S because of its cytoprotective and vasodilating activity is an attractive therapeutic candidate to decrease the destructive effects of proteinuria and hypertension (15). 

## 2. Objectives

 The study presented here attempted to evaluate the nephroprotective effects of the NaHS (an H2S-releasing molecule) on CP-induced renal injury. 

## 3. Materials and Methods

###  3.1. Animals 

 Thirty-two male healthy Sprague-Dawley rats (180 ± 20) were obtained from animal house of Ahvaz Jundishapur University of Medical Science (Ahvaz, Iran). Rats were maintained under standard conditions of temperature (21 ± 2) and exposed to a 12-hour dark-light cycle and allowed free access to a standard pellet diet and water. 

###  3.2. Reagents and antibodies 

 Cisplatin, NaHS and other chemicals and reagents were obtained from Sigma Aldrich chemical Co. (St. Louis, USA). Antibodies against nephrin and desmin were purchased from Santa Cruz Biotechnology (Santa Cruz, CA). 

###  3.3. Experimental design 

 All rats were randomly divided into 4 groups (each n= 8): normal control group (A group) was injected with saline‚ NaHS group (B group) was injected with 200 µg/kg/d (intraperitoneal injection;i.p.) for 15 days‚ CP group (C group) was given one doses of 5mg/kg i.p., and CP + NaHS group (D group) were injected with one doses of CP (5 mg/kg i.p) and then NaHS (200 µg/kg/d i.p.) for 15 days. A pilot study was performed to determine effective doses of NaHS at 50, 100 and 200 µg/kg/d dosages. On the 15 day, to determination of 24-hour urine protein (UP) excretion, urine samples were collected one day prior to sacrifice, the animals were sacrificed under ketamine anesthesia then, blood samples were obtained to detection of blood urea nitrogen (BUN) and creatinine (Cr) levels. Also, their right kidneys were rapidly removed and were fixed in formalin (10%) and left kidneys were frozen immediately in liquid nitrogen. 

###  3.4. Biochemical analysis 

 Biochemical parameters in serum and urine were determined by auto analyzer (Vita lab Selectra E, Netherland) by commercially available kits (Pars Azmon, Iran). 

###  3.5. Histopathology 

 After fixation, Specimens were processed for paraffin embedding and three µm sections were prepared. Sections were stained with periodic acid–Schiff (PAS) and were observed under light microscope for any histopathological changes. 

###  3.6. Estimation of malondialdehyde in kidney tissue 

 The kidneys were homogenized in potassium phosphate buffer (10 mM, pH 7.4) at a concentration of 5% (w/v) using a homogenizer (Model silent crusher-M;Heidolph Instruments, Donau, Germany). The homogenates were centrifuged at 16 000× g at 4°C for 20 minutes. Levels of malondialdehyde (MDA), was determined colorimetrically by thiobarbituric acid reactive substances (TBARS) as described in previous study (16). 

###  3.7. Immunohistochemistry analysis 

 Immunohistochemistry was carried out as explained previously (17). Briefly‚ kidney sections (5 µm in thickness) were deparaffinized by xylene and dehydrated in graded concentrations of ethanol and for antigen retrieval, sections were pretreated into 10 mM citrate buffer (pH 6.0) and heated with a microwave for 15 minutes. After that, sections were incubated with 1% H_2_O_2_ for 20 minutes to inactivate endogenous peroxidase and were washed twice in PBS and then blocked with 1.5% blocking serum in PBS for 1 hour. They were then incubated with primary antibody (goat polyclonal anti-desmin at the dilution 1:200), overnight at 4℃, washed with PBS, incubated for 60 minutes with the secondary biotinylated antibody (biotinylated anti-goat IgG). After washing twice in PBS, antibody location was revealed using diaminobenzidin as peroxidase substrate. Color development was stopped by washing in water. Sections were counterstained with Meyer’s hematoxylin, dehydrated and mounted. Negative controls were performed using normal serum instead of primary antibody. 

###  3.8. Western blotting 

 Western blotting was carried out as explained in our previous study (18). The renal cortex was homogenized in lysis buffer (150 mM NaCl, 0.25% wt/vol sodium deoxycholate, 50 mM Tris-HCl [pH 7.5], 1% Triton X-100, 0.1% SDS, 1mM EDTA, 1% protease inhibitor cocktail [Roche, Mannheim, Germany]) using an ultrasonic homogenizer on ice. Homogenates were centrifuged at 13 000 × g at 4°C for 20 minutes and the supernatants were collected and the protein concentration of them were determined using the Bradford method. Then 150 µg of total protein were separated by 10% SDS-PAGE and electro transferred to PVDF membrane. Nonspecific binding was blocked with 5% bovine serum albumin in TBS for 1h at room temperature. Following washing with 0.02% Tween-20/TBS (TBST), the membranes were incubated overnight at 4°C with the primary antibody: nephrin, and β-actin, (1:200 dilution). The membranes were washed with TBST and incubated for 1 hour at room temperature with HRP conjugated secondary antibodies (nephrin was donkey anti-goat IgG and β-actin was goat anti-rabbit IgG). Visualization was performed using ECL kit (Najm Biotech ECL, Iran). 

###  3.9. Ethical issues 

 The research followed the tenets of the Declaration of Helsinki. This project were approved by Ethics Committee of Ahvaz Jundishapur University of Medical Sciences. Prior to the experiment, the protocols were confirmed to be in accor­dance with the guidelines of Animal Ethics Committee of Jundishapur University of Medical Sciences. 

###  3.10. Statistical analysis 

 SPSS version 15.0 for Windows was used for all analyses. Data were expressed as means ± SD. Differences between groups were tested with the use of one-way analysis of variance (ANOVA) with post hoc Tukey’s test. Significance was accepted at *P* < 0.05. 

## 4. Results

###  4.1. Body and kidney weight and urine volume 

 The body weight of rats in C group was significantly less that of rats in A and B groups (*P* < 0.001). But, the kidney weight and urine volume of rats in C group was significantly more than rats of A and B groups (*P* < 0.01) whereas administration of NaHS in D group significantly improved this reduction of body weight (*P* < 0.01) and also, increased kidney weight and urine volume (*P* < 0.05) when compared with rats in C group (Table 1). 

**Table 1:** Effect of cisplatin and NaHS on body weight, kidney weight and urine volume

**Group**	**Body weight (g)**	**Kidney weight (g)**	**Urine volume (mL/24 h)**
A	177.00 ± 21.83	0.82 ± 0.13	0.85 ± 0.14
B	190.50 ± 28.85	0.89 ± 0.08	1.25 ± 0.27
C	144.83 ± 16.70*	1.00 ± 0.16*	4.50 ± 1.04*
D	169.80 ± 17.08^#^	0.81 ± 0.10^#^	2.24 ± 0.15^#^

Data are expressed as mean ± SD; Normal control group (A), NaHS group (B), Cisplatin group (C), CP + NaHS group (D), ^*^P < 0.01 compared with A and B groups, ^#^*P* < 0.01 compared with the CP-treated group (C).

###  4.2. Serum and urine biochemical analysis 

 As shown in Table 2, serum Cr and BUN as well as 24-hour UP in C group significantly increased (*P* < 0.01) following CP injection. Treatment with NaHS reduced levels of Cr, BUN and 24-hour UP in D group when compared with C group (*P* < 0.01). 

**Table 2:** Effect of NaHS and Cisplatin on biochemical indicators.

**Group**	**Creatinine (mg/dL)**	**Urea** **(mg/dL)**	**Urine protein (mg/24 h)**
A	0.57 ± 0.09	15.25 ± 3.01	0.60 ± 0.12
B	0.56 ± 0.08	20.00 ± 3.20	0.71 ± 0.16
C	0.80 ± 0.11*	28.00 ± 6.60*	5.50 ± 1.54*
D	0.60 ± 0.1^#^	15.80 ± 2.60^#^	2.81 ± 1.10^#^

Data are expressed as mean ± SD; Normal control group (A), NaHS group (B), Cisplatin group (C), CP + NaHS group (D), ^*^P < 0.01 compared with A and B groups, ^#^*P* < 0.01 compared with the CP-treated group (C).

###  4.3. Histopathological findings 

**Figure 1:**
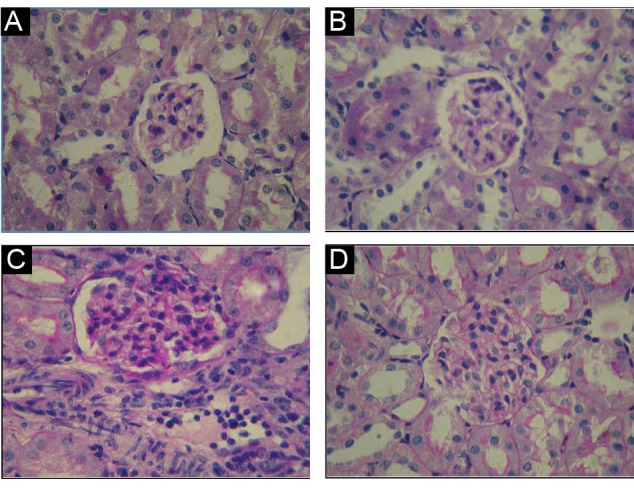
Effect of NaHS on cisplatin (CP)–induced histopathological alterations in renal tissues of different groups. Normal control group (A), NaHS group (B), cisplatin group (C), CP + NaHS group (D), (PAS staining ×400).

 As indicated in Figure 1, under the light microscopic evaluation, no obvious pathological changes were found in the kidney of A and B groups whereas treated group with CP (C group) showed degenerative changes in kidney include increase of mesangial matrix and proliferation of glomerular mesangial cells. In D group these pathological damages were very lighter than those of C group. 

###  4.4. Renal MDA level 

**Figure 2:**
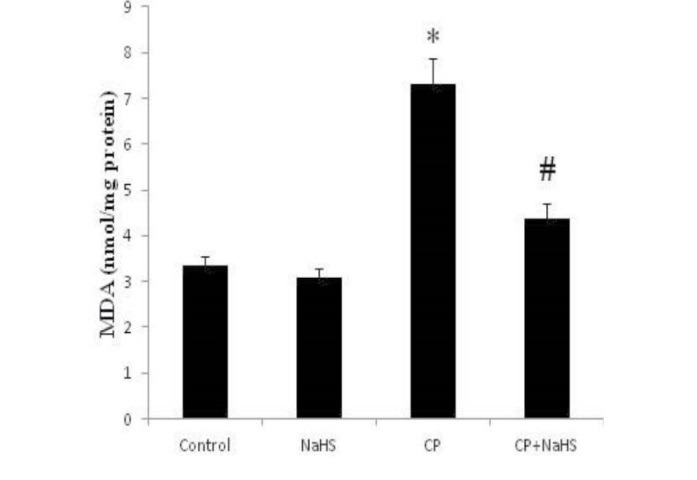
Effects of NaHS on CP- induced on MDA level alterations in renal tissue of different groups. Data are expressed as mean ± SD, *P < 0.001 compared to A and B groups, # P < 0.001 compared to CP-treated group (C).

 The levels of MDA in renal tissue were higher in C group than the******A and B groups (*P* < 0.001), treatment with NaHS in D group could reverse (*P* < 0.001) this increase (Figure 2). 

 In all the above parameters there was no significant difference between groups of A and B (*P* > 0.05). 

###  4.5. Immunohistochemistry for desmin protein 

**Figure 3:**
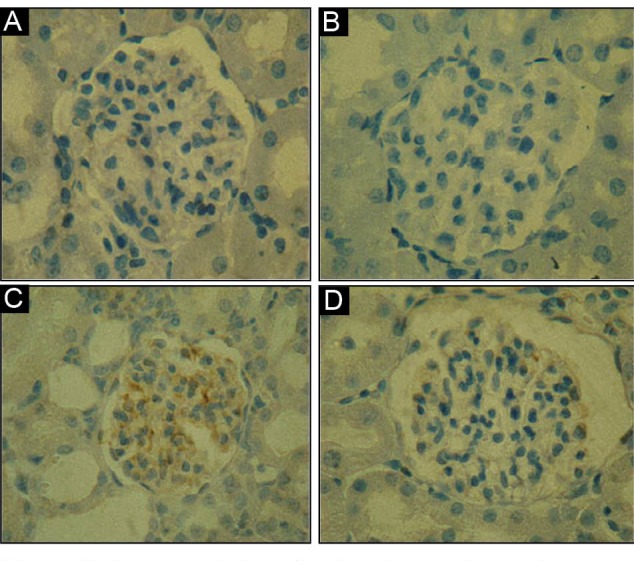
Immunostainings for desmin protein are shown on tissue sections. Desmin was detected only in glomeruli of different groups; Normal control group (A), NaHS group (B), cisplatin group(C), CP + NaHS group (D) (×400).

 The renal cortex of rats in A and B groups demonstrated a negative desmin immunostaining in the renal glomerulus, indicating which no podocyte cytoskeleton injury (Figure 3A and  3B). C group following administration of CP indicated positive desmin immunostaining in the renal glomerulus indicating that a notable podocyte injury (Figure 3C) while D group after administration of NaHS indicated decline of desmin immunostaining in the renal glomerulus (Figure 3D). 

###  4.6. Western blotting for nephrin protein 

**Figure 4:**
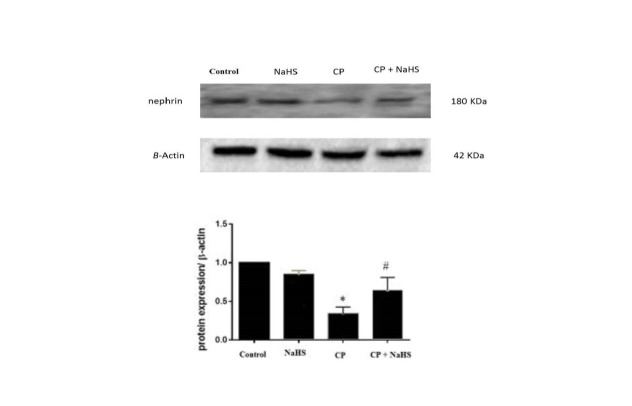
Effects of NaHS on nephrin protein expressions in different groups. Western blot showed that the CP group had lower expressions. In addition, compared with CP group, nephrin expression was up-regulated after NaHS treatment. Data were expressed as means ± *P* < 0.05 compared with the normal control and NaHS groups, # *P* < 0.05 compared with CP group.

 As shown in Figure 4, protein level of nephrin was significantly decreased in CP-treated group (C group) compared with the A and B groups (*P* < 0.05). In contrast, NaHS treatment increased the protein levels of nephrin in D group (*P* < 0.05). 

## 5. Discussion

 In the present study, NaHS (a bioactive compound releasing H_2_S) showed protective effects on CP-induced renal lesions in rats. In this study, single injection of CP (5 mg/kg i.p) showed a significant reduction in the body weight and a remarkable increase in the weight of the kidneys as well as urine volume. Previous studies are in agreement with our results (19). The body weight loss in rats treated with CP was possibly because CP has a direct toxic effect on renal tubules that resulted reduced reabsorption of water and excretion of excessive sodium with subsequent polyuria, dehydration and body weight reduction;or might be because of gastrointestinal toxic effects including reduction in appetite, ingestion and assimilation of food (20,21). Moreover, increase in kidney weight might be due to the edema of renal parenchyma since CP was known to cause renal inflammation (22,23). The body and kidney weight and also, urine volume is returned by NaHS treatment close to the control group. This effect approved that NaHS is possibly effective for protecting against the toxic effects of CP on renal. The present research has shown that CP enhanced concentrations of serum creatinine and BUN as well as 24-hour UP. The possible reasons of this increase in biomarkers of renal in serum and urine were the impaired functions of renal, obstruction of tubular, and/or the back-leakage of the renal tubules (23). Such functional disturbance in rats treated with CP could indicate the CP ability in protein synthesis inhibition in the tubular cells or lipid peroxidation initiation and free radicals generation in renal tubules (22,24). These results were obtained in several previous studies (25,26). However, the concentrations of serum BUN and creatinine as well as 24-hour UP showed marked progress in rats treated with the NaHS + CP (D group). These outcomes proved the NaHS ability for improving or normalizing the function of renal. H_2_S contributes in controlling the function of renal and enhances excretion of urinary sodium through both renal vascular and tubular actions (10,27). Protective activity of H_2_S is related to the inhibition of ROS production through inhibiting NADH oxidase, inducting anti-oxidative molecules such as, thioredoxin, and also increasing production of glutathione (GSH) (28). Moreover, anti-inflammatory effects of NaHS is probably reason of urinary protein reduction, thereby preserving the endothelial barrier in the glomeruli (29). The results of current study are in accordance with the results of earlier research (21). In the current study, the main histopathological results of renal tissues treated with CP were the extension of mesangial matrix, the tubular epithelium degeneration and the interstitial inflammatory cell infiltration. These observations agreed with those of the earlier research (30,31). Though, the evaluation of renal histopathological in groups treated with NaHS also showed a reduction in structural changes induced by CP. However, no clear nephro-protective mechanism has been understood for NaHS, there is evidence on strong antioxidant activity of NaHS in some organs (32,33). ROS formation in kidney has a key role in renal injury induced by CP, and different thiol compounds and antioxidants have been presented for protecting against CP-induced renal damage (11,33). As mentioned, CP-induced renal injury is associated with the generation of ROS, MDA and a decrease in antioxidant systems in kidney and NaHS because of its antioxidant properties can reduce oxidative stress (28,30). Thus, these confirm our result about MDA. In addition, our results demonstrated that CP caused an increase in the desmin expression and a reduction in the expression of nephrin. It has been proposed that CP could enhance the production of free radical and the stress of oxidative (33,34). In many models of podocyte injury, enhanced production of ROS has been observed (4). An increase in proteinuria is caused by podocyte injury. Furthermore, the podocyte injury confirmed reduced nephrin expression (glomerular SD protein) and enhanced desmin expression (an intermediate filament protein of the cytoskeleton and podocyte injury marker) (35). The structure and the function of podocytes are affected by proteins of cytoskeletal and SD. In the current research, NaHS enhanced the nephrin expression and reduced expression of desmin in rats with CP-induced renal injury. In general, the current research proposes that protection of NaHS against renal injury was possibly accomplished via changing foot process cytoskeleton and expression of SD protein component, causing alliterating structure in podocytes and consequently restoring normal morphology and repairing the filtration barrier of glomerular. Although in this study, we have showed that the alternative expressions of nephrin and desmin proteins are associated with the podocyte injury, but their roles in the occurrence and development proteinuria has been not known clearly. Further studies are needed for other mechanisms underlying the NaHS therapeutic influence on podocytes in renal damage. 

## 6. Conclusions

 The present study highlights the potential role of NaHS in alleviating CP-induced renal injury via reducing histological damages, desmin protein expression, 24-h UP, urine volume, Serum Cr, BUN and MDA and also, via increasing nephrin protein expression. 

## Acknowledgments

 This work was a part of MSc student thesis that supported by the Ahvaz Jundishpur University of Medical Sciences (Grant # CMRC-9421). 

## Conflicts of interest

 The authors declare that they have no competing financial interests in relation to the work described. 

## Authors’ contribution

 AK;participated in the performance of the research. FA;contributed to all aspect of the study. LKH;achieved statics and data analysis. AV;collected the data. EM;participated in research design and the writing of the paper. 

## Funding/Support

 This study was supported by Deputy of Research and Technology Development Ahvaz Jundishpur University of Medical Sciences. 
